# Determinant factor of married women’s knowledge on vertical transmission of HIV in Mecha district, Ethiopia; a community-based study

**DOI:** 10.1371/journal.pone.0242659

**Published:** 2020-12-02

**Authors:** Tewachew Muche Liyeh

**Affiliations:** Department of Midwifery, College of Health Sciences, Debre Tabor University, Debre Tabor, Ethiopia; 1. IRCCS Neuromed 2. Doctors with Africa CUAMM, ITALY

## Abstract

**Introduction:**

Mother-to-child transmission (MTCT) of HIV infection remains a major public health problem and constitutes the most important cause of HIV infection in children. Knowledge of married women on MTCT of HIV is very important for successful intervention toward prevention of mother-to-child transmission (PMTCT) and achieving the goal of eliminating the new HIV infection. The objective of the study was to assess knowledge of married women on MTCT of HIV and associated factors in Mecha district Northwest Ethiopia.

**Methods:**

A community based cross sectional study was conducted involving 520 married women from January 1 to February 30 /2017 in Mecha district. Interviewer administered questionnaires were used to collect the data. The collected data was entered, cleaned and checked using Epi Data version 3.1 and then analyzed with SPSS version 20. Bivariate and multivariable logistic regression was computed for all explanatory variables to identify determinant factors at 95% confidence interval. Explanatory variables having P-value <0.05 taken as a predictor for knowledge of married women on mother to child transmission of HIV.

**Result:**

This study was carried out among a total of 520 married reproductive age group women with a response rate of 98%. From the total of 510 respondents, 160(31.4%) of women were knowledgeable on vertical transmission of HIV (MTCT). Women who were knowledgeable on MTCT of HIV was positively associated with urban residence (AOR = 1.75, 95% CI: 1.05–2.92), women who had history of ANC follow up (AOR = 2.68, 95% CI: 1.17–6.13), women who were pregnant during the study period (AOR = 1.86, 95%CI: 1.10–3.13) and those who had discussions with their husband about HIV/AIDS/ MTCT (AOR = 2.40, 95% CI: 1.52-.3.80).

**Conclusion and recommendation:**

The finding from this study revealed that knowledge of married women on MTCT of HIV was low. This may contribute to increase the spread of MTCT of HIV. Therefore, giving more attention and emphasis on continuous education regarding MTCT of HIV is highly recommended.

## 1. Introduction

Since the beginning of the epidemic, around 75.7 million people have been infected with the HIV virus and about 35 million people were died. Globally, 32.7 million people were living with HIV at the end of 2019 [1]. Sub-Saharan Africa remains most severely affected, with 4.4% of adults living with HIV and accounting for nearly 70% of the people living with HIV worldwide [2]. In Ethiopia about 690 000 people were living with HIV in 2018 of whom 23 000 people were newly infected and the prevalence of HIV among women aged 15 to 49 were 1.2% [3].

One of the consequences of HIV infection in married reproductive aged women is vertical transmission of the virus to their children. Mother-to-child transmission (MTCT) of human immune deficiency virus (HIV) infection is the transmission of the virus from an HIV-infected mother to her child during pregnancy, labor, delivery or breastfeeding. Mother-to-child transmission (MTCT) of HIV infection leftovers the central public health problem and constitutes the most important cause of HIV infection in children [4].

Without any intervention, the risk of a baby getting HIV infection from an infected mother is estimated to be 20% to 45% (5% to 10% during pregnancy, 10% to 15% during delivery and 5% to 20% through breast- feeding) [5]. Globally, approximately 880 children became infected with HIV and approximately 310 children died from AIDS related causes in each day in 2019. Most of the children infected with HIV were due to mother-to-child transmission [6].

In 2011, the government of Ethiopia adopted option A to provide more effective ARV drugs to reduce mother-to child transmission of HIV then the strategy shifts to option B+ in August 2012 [7]. However, mother-to child transmission (MTCT) of HIV has remained a challenge for the country; Ethiopia [8]. Recent reports in 2016 revealed that, in Ethiopia about 109,133 children less than 15 years were living with HIV in and there are an estimated 2,420 new infections each year due to vertical transmission [9]. Recent study done in Ethiopia revealed that almost ten percent of HIV exposed infants become HIV positive [10]. The availability of PMTCT service by itself doesn’t improve women’s PMTCT utilization. For PMTCT to be successful, married reproductive age women should be knowledgeable on period of vertical transmission HIV infection.

Even though there were some studies conducted at institution level, little is known about determinant factor of married women’s knowledge on vertical transmission of HIV at the community level in Ethiopia generally and in the study area particularly. Therefore, this study was conducted to fill these gaps.

## 2. Methods

### 2.1. Study design and setting

A Community based cross sectional study was conducted from January 1 to February 30 /2017 in Mecha district. Mecha district is located in Amhara regional state, North West part of Ethiopia which is 531 km from Addis Ababa, the capital city of Ethiopia. There are six Urban and 40 rural kebeles (the smallest unit of the district) in the district. According to the 2016 central statistics agency report, the total populations of the district were 383,861. The main source of income for Mecha district community is mixed agriculture. Amharic is the official language of the community. The district has one general hospital, six health center and four private clinics.

### 2.2. Participants

#### 2.2.1. Source population

All married women living in Mecha district were the source population of the study.

#### 2.2.2. Study population

Those married reproductive age women living in Mecha district during constituted as the study population.

#### 2.2.3. Study unit

Individual married reproductive age group woman was the study unit.

#### 2.2.4 Eligibility criteria

All married reproductive age group women who lived at least for 6 months in the study area.

### 2.3. Sampling technique and procedure

Sample size was calculated by using single population proportion formula considering 95% level of confidence, 19% of the respondents were knowledgeable on MTCT of HIV/AIDS taken from another study, 5% of margin of error and design effect of two. Finally, considering a non-response rate of 10%, the total sample size was 520.

To select the study participants, multi-stage sampling technique was used. First kebeles were divided in to rural and urban kebeles. Then from 46 kebeles 10 rural and 2 urban kebeles were randomly selected by lottery method. Then individual households in the chosen kebeles were selected by using a systematic sampling technique. The first household for each selected kebele were selected by lottery method starting from Northeast direction and every 22^nd^ house for all kebele was asked. The sampling interval of the households in each kebele was determined by dividing the total number of households in the specific kebele by the allocated sample size. In the case of more than one eligible participant in the household, lottery method was used to select only one. Respondents who were unable to communicate were excluded.

### 2.4. Operational definition

#### 2.4.1. Knowledge on MTCT

If the respondents answered correctly all the three period of MTCT of HIV (during pregnancy, during delivery, and through breastfeeding) categorized as knowledgeable and non-knowledgeable if they answered less than three questions.

### 2.5. Data collection tools and techniques

An interviewer-administered questionnaire was developed for the purpose of data collection after reviewing relevant literatures [10–19]. The questionnaire was first developed in English and transferred to local language (Amharic) then back to English to keep its consistency by language experts. The data were collected by six diploma holding midwives after two days training about informed consent, techniques of interviewing, data collection procedures, and different sections of the questionnaire. Two health officers were assigned as supervisors for the data collectors and overall supervision also made by the principal investigators. Before starting the actual survey, the questionnaire was pre-tested on 5% (26) individuals outside of the district and the necessary modification on the questionnaire and data collection procedures made. The collected data was reviewed and checked for completeness before data entry; the incomplete data was discarded. Data entry format template was produced and programmed.

### 2.6. Data analysis

First, the collected data were entered, cleaned, and checked by the Epi Data software version 3.1 and then exported to SPSS version 20 for analysis. Descriptive statistics was done to describe the study population in relation to relevant variables by using tables and graphs. Bivariate analysis was computed for all explanatory variables in relation to knowledge on MTCT of HIV, and those predictor variables having P value < 0.2 were entered in to multivariable logistic regression for analysis adjustment of confounding effect between explanatory variables. Adjusted odds ratio with 95% confidence interval was computed and P-value less than 0.05 considered a predictor variable for knowledge of married women on MTCT of HIV. The model was checked by using Hosmer and Lemeshow test of fitness.

### 2.7. Ethical considerations

Ethical clearance and approval were obtained from Ethical Review Board of University of Gondar and letter of cooperation has been received from Mecha woreda health office. Then the Participants of the study were informed about the purpose of the study, the importance of their participation, and their right to withdraw at any time. Written informed consent was also obtained from each participant prior to data collection. Confidentiality of the information was maintained throughout by using anonymity identifiers, keeping their privacy by interviewing them individually.

## 3. Result

### 3.1. Socio-demographic characteristics of the participants

This study was carried out among a total of 520 married reproductive age group women with a response rate of 98%. The mean (+ SD) age of respondents was 26.9 ± 7.4 years. Majority 377 (73.9%) of the study population were from rural area. Most of the study participants 488 (95.7%) were followers of orthodox Christianity in religion. About 409 (80.2%) of the respondents had no formal education and 447 (87.6%) of the study participants were house wives. Four hundred fifty-one (84.5%) of the participants had the accessibility of health facility within 5 kilometers (Table 1).

**Table 1:** Socio-demographic characteristics of married women in Mecha district, North west Ethiopia, 2017 (N = 510).

Variables	Category	frequency	Percent (%)
Age	20–30	148	29
	31–40	74	14.5
	>40	288	56.5
Residence	Rural	377	73.9
	Urban	133	26.1
Religion	Orthodox	488	95.7
	Muslim	22	4.3
Educational status	no formal education	409	80.2
	primary education	24	4.7
	secondary and above	77	15.1
Husband’s educational status	no formal education	437	85.7
	primary education	25	4.9
	secondary and above	48	9.4
Occupation	house wife	447	87.6
	Gov't employee	25	4.9
	market trade vender	16	3.1
	daily laborer	22	4.3
Husband’s occupation	Farmer	387	75.9
	Gov’t employee	27	5.3
	Daily laborer	43	8.4
	Market trade vender	31	6.1
	Other*	22	4.3
distance from health institution	0.1–5 km	451	84.5
	>5 km	59	15.5

Others

*: religious leaders.

### 3.2. Obstetrics characteristics of the respondents

Among a total of 510 participants, 230 (45.4%) were multigravida. Around one fifth of the respondents were pregnant at the time of the study. Three hundred seventy-three (82.5%) and 288 (63.7%) of women had history of ANC follow up during the last pregnancy and had history of institutional delivery respectively. Majority 438 (85.9%) of the respondents had history of using family planning (Table 2).

**Table 2:** Obstetrics characteristics of married women in Mecha district, North West Ethiopia, 2017 (N = 510).

Variables	Category	frequency	percent
Gravidity	Nuli gravida	58	11.4
	Primi gravida	79	15.5
	Multi gravida	233	45.7
	Grand multi	140	27.5
	Total	510	100.0
current pregnancy status	Yes	104	20.4
	No	406	79.6
	Total	510	100.0
history of ANC visit	Yes	373	82.5
	No	79	17.5
	Total	452	100.0
history of institutional delivery	Yes	288	63.7
	No	164	36.3
	Total	452	100.0
history of family planning use	Yes	438	85.9
	No	72	14.1
	Total	510	100.0

### 3.3. Knowledge of the married women on HIV/AIDS and MTCT

All of the respondents had heard about HIV/AIDS. More than three fourths, 390 (76.5%) of the women had knew their sero-status. In this study 160 (31.4%) of the respondents were knowledgeable on MTCT of HIV. Respondents were further asked to identify the sources of information and health professionals were the most frequently mentioned source of information, 402 (78.8%) followed by from friends and relatives 229 (44.9%) (Table 3).

**Table 3:** Knowledge of HIV/AIDS and MTCT among married women in Mecha district, North West Ethiopia, 2017.

Variables	Category	Frequency	Percent
Heard HIV/AIDS	yes	510	100
Ever tested for HIV	yes	390	76.5
	no	120	23.5
	Total	510	100
Had discussion with their husband about HIV/AIDS	yes	187	36.7
	no	323	65.3
	Total	510	100
Knowledge on mode of HIV transmission	By unprotected sex with un infected person	267	52.4
	By blood transfusion with infected blood	33	6.5
	By sharing sharp instruments	425	83.3
	By mosquito bite	108	21.2
	Through MTCT	410	80.4
Knowledge of respondents on MTCT	Knowledgeable	160	31.4
	Non knowledgeable	350	68.5
Source of information on HIV/AIDS	Health professionals	402	78.8
	Friends/relatives	229	44.9
	school	106	20.8
	multimedia	70	13.7

**Fig 1:**
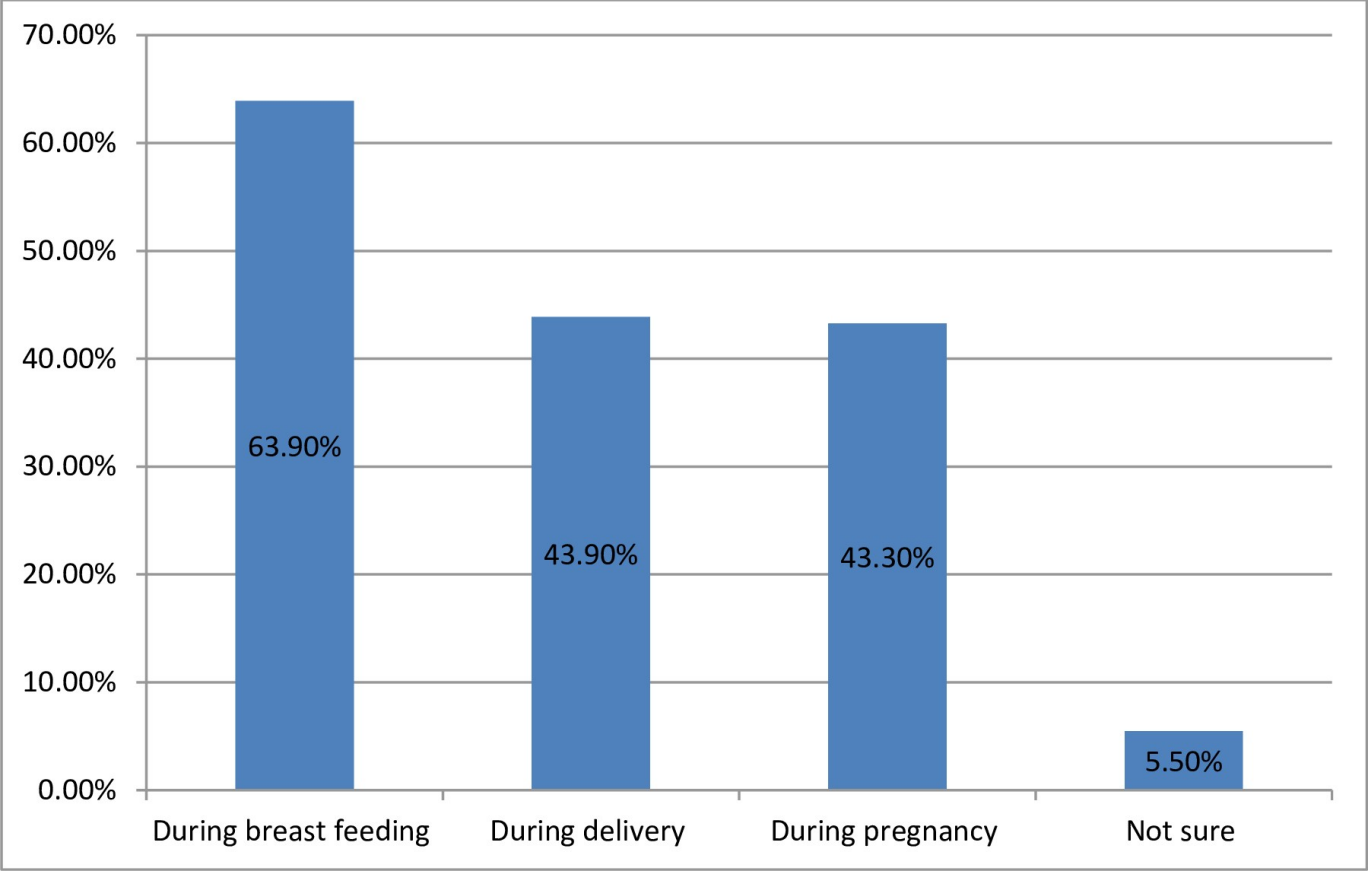
Knowledge of married women on period of MTCT in Mecha district, North West Ethiopia, 2017, (N = 410).

Regarding the period of vertical transmission of HIV, Four hundred ten (80.4%) knew that HIV can be transmitted from an infected mother to her baby of whom, 326 (63.9%) responded that MTCT of HIV could be occurred through breast feeding and 23 (5.5%) didn’t know the exact period of HIV transmission (Fig 1).

### 3.4. Factors associated with knowledge of married women on vertical transmission of HIV

In bivariate analysis residence, history of ANC follow up, history of institutional delivery, history of using family planning, women who were pregnant, women who had discussions with their husband about HIV/AIDS and women who knew their sero-status were significantly associated with women’s knowledge on MTCT and entered to multivariable logistic regression for adjustment of confounding effect between explanatory variables. Finally, in multivariable logistic regression model only residence, those who had history of ANC follow up, women who were pregnant during the study period and women who had discussions with their husband about HIV/AIDS/MTCT were significantly associated with the outcome variable.

Urban dweller women were 1.75 times more likely (AOR = 1.75, 95% = CI = 1.05–2.92) to be knowledgeable on MTCT of HIV than rural counterparts. Respondents who had history of ANC follow up were about 2.68 times (AOR = 2.68, 95% CI = 1.17–6.13) more knowledgeable on MTCT of HIV/AIDS than those who did not have. Women who were pregnant during the study period were 1.86 times more likely (AOR = 1.86, 95%CI = 1.10–3.13) to be knowledgeable than those who were not pregnant. Women who had discussions with their spouse about HIV/AIDS/ MTCT were 2.4 times (AOR = 2.40, 95% CI = 1.52-.3.80) more likely to have better knowledge on MTCT than those who had not (Table 4).

**Table 4:** Factors associated with knowledge of MTCT of HIV among married women in Mecha district, North West Ethiopia, 2017.

Variables	category	Knowledgeable on MTCT		COR (95%CI)	AOR (95%CI)	P-value
		Yes	no			
Residence	urban	61	72	2.87 (1.89–4.35)	1.75 (1.05–2.92)	0.033**
	rural	86	291	1	1	
History of FP utilization	yes	139	299	3.72 (6.44–15.59)	2.70 (0.96–7.63)	0.061
	no	8	64	1	1	
history of ANC visit	yes	119	254	3.23 (1.60–6.49)	2.68 (1.17–6.13)	0.020**
	No	10	69	1	1	
history of institutional delivery	yes	103	185	2.96 (1.82–4.79)	0.96 (0.50–1.82)	0.90
	no	26	138	1	1	
Currently pregnant	yes	44	60	2.16 (1.38–3.38)	1.86 (1.10–3.13)	0.020**
	no	103	303	1	1	
Discussion with husband about HIV /MTCT	yes	76	111	2.43 (1.64–3.60)	2.40 (1.52-.3.80)	<0.0001**
	no	71	252	1	1	
Ever tested HIV	yes	123	267	1.84 (1.122–3.03)	0.97 (0.54–1.77)	0.95
	no	24	96	1	1	

** = P-value <0.05.

## 4. Discussion

Improving knowledge on period of MTCT of HIV among populations at higher risk of HIV infection has great importance particularly in the reduction of childhood and maternal morbidity and mortality which in turn has enormous impact on socioeconomic development of the country.

In this study, all of the respondents heard about HIV/AIDS. This finding was the consistent with studies done in Addis Ababa at Tikur Anbessa and Zewuditu memorial hospitals, Hawasa university hospital, Ambo hospital and southern Ethiopia [11–14]. This may be because of the universal nature of the problem and expansion of mass media to get information easily.

Nearly one third, 160 (31.4%) of the participants were knowledgeable on MTCT of HIV. The finding of this study is higher than the studies done in Meket District (19%) [15] and Southern Ethiopia (11.5%) [14]. This could be due to the difference in the study setting and accessibility of health facilities. However, this result is lower than studies conducted in Debre Markos town (42.3%) [16], Asosa, Ethiopia (57.5%) [17] and Cameroon (37%) [18]. This discrepancy might be due to the study setting and source population. This study is community based with source population of all married women whereas the study in Debre Markos, Asosa and Cameroon used institutional based study among ANC attendees who have more accessible for MTCT information.

Knowledge of reproductive age women on MTCT of HIV was significantly varied based on their place of residence. Those women living in urban areas were 1.75 times (AOR = 1.75, 95% = CI = 1.05–2.92) more likely to be knowledgeable when compared to the rural counter parts. This finding is in line with studies conducted at Hawassa Referral Hospital, Meket district and Tanzania [11, 15, 20]. It might be due to women living in urban setting are more likely to be educated and might have better access to print media exposure than those women living in rural areas.

Positive association was reported between women having knowledge on MTCT of HIV with history of ANC follow up. Women who had history of ANC follow up were about 2.68 times more likely (AOR = 2.68, 95% CI = 1.17–6.13) to be knowledgeable on vertical transmission of HIV/AIDS than those who did not have. This finding is in line with the study conducted at Hawassa referral hospital [11]. It could be due to women who had ANC follow up might get the chance to learn from health care providers and this information may enhance women’s knowledge about mother to child transmission of HIV. Women who were pregnant during the study period were 1.86 times (AOR = 1.86, 95%CI = 1.10–3.13) more likely to be knowledgeable than those who were not pregnant. It could be due to pregnant women might have the probability of getting information during their ANC follow up.

Women’s knowledge on period of MTCT was positively associated with having a discussion about MTCT with their male partner. Women who had discussions with their husband about MTCT and were 2.7 times (AOR = 2.700, 95%CI = 1.658, 4.396) more likely to be knowledgeable than those who had not. This finding is in line with the study done on southern Ethiopia and Mekele and Malawi [14, 21, 22]. This might be explained due to couples’ communication regarding HIV/AIDS and MTCT will help to share the information and increase their level of understanding which enhances their knowledge about MTCT.

This study has its own strength and limitation. The first strength was since the study was community based, it can be inferred the entire population at large. The other strength was the sample size which was relatively large. As a limitation, being cross-sectional study might make difficult to draw conclusion about cause effect relationship.

## 5. Conclusion

The result of this study verified that knowledge of MTCT of HIV among married women was found to be low. This may contribute to increase transmission of MTCT of HIV. Residence, those having a history of ANC follow up, Women who were pregnant during the study period, women who held discussions with their husband about HIV/AIDS/MTCT were significantly associated with women’s knowledge on MTCT. Therefore, giving more attention and emphasis on continuous education regarding MTCT of HIV is highly recommended.

## Supporting information

S1 File English version questionnaire used to collect data from the women. (DOCX)

S2 File Amharic version questionnaire used to collect data from the women. (DOCX)

## List of abbreviations

AIDS: Acquired Immunodeficiency Syndrome
AOR: Adjusted Odds Ratio
ART: Anti-Retroviral Therapy
CI: Confidence Interval
EDHS: Ethiopian Demographic and Health Survey
HIV: Human Immunodeficiency Virus
MTCT: Mother to Child Transmission
PMTCT: Prevention of Mother to Child Transmission
WHO: World Health Organization
