# Supplementary material for: Determinant factor of married women’s knowledge on vertical transmission of HIV in Mecha district, Ethiopia; a community-based study
Source: PLoS One. 2020 Dec 2;15(12):e0242659. doi: 10.1371/journal.pone.0242659 (PMC7710110; doi:10.1371/journal.pone.0242659)
Supplement: S1 File — (DOCX) [file pone.0242659.s001.docx]

***Information Sheet and consent form***

Hello! My name is -------------I am here today to collect data on determinant factor of married women’s knowledge on vertical transmission of HIV. You are selected as a participant for this investigation and before getting your permission for participation, let me give you all the necessary information regarding the study.

**Purpose of the study**:

To assess determinant factor of married women’s knowledge on vertical transmission of HIV in Mecha district, Ethiopia, 2017.

**Procedure**:

There are questions to assess determinant factor of married women’s knowledge on vertical transmission of HIV. I would like to ask you to give your genuine and honest answers on the questions will be forwarded. If you need clarification please ask me. The interview will take about 20 minutes to finish.

**Benefit of the study:**

For being involved in this study, there is no payment and special privilegeyou. Perhaps, participating and giving information for the questions being asked plays a pivotal role to address the gap; mainly to identify factors influencing knowledge of MTCT in Mecha district. **Risk**: There will be no physical or psychological harm during the procedure. Besides, you have full right to stop any time you wish and you won’t be obliged to give any information which you don’t want to answer.

**Confidentiality**:

Any information you give will be kept confidential and won’t be accessible to any third party. Your name won’t be mentioned anywhere. The information you give is only used for research purpose and will be burnt at the end.

your participation in this study will totally be on the basis of your willingness. You can stop anywhere you wish to stop participation, even from the very beginning. No one will force you to give information you don’t want to give. Finally, I duly acknowledge your participation and either response.

Are you volunteer to participate in this study

Yes No Signature_______________

Datacollector: Name___________Signature_______________Date_________

Principal investigator: Tewachew Muche Signature __________, date ___________.

Contact Address: Cell phone: 0918475767 Email : [tewye2006@gmail.coml](mailto:tewye2006@gmail.coml)

English version questionnaire:

Instructions: Encircle the possible answer (more than one answer is possible) and write the required responses in the spaces provided

| **O1. Socio-demographic characteristics** | | | |
| --- | --- | --- | --- |
| N0. | QUESTIONS | RESPONSE | remark |
| 101 | How old are you? | _________ | Complete years |
| 102 | What is your residence | 1. Urban 2. Rural |  |
| 103 | What is your educational status? | 1. No formal education 2. Primary education 3. Secondary and above |  |
| 104 | What is the educational status of your husband? | 1. No formal education 2. Primary education 3. Secondary and above |  |
| 105 | What is your current occupation? | 1. House wife 2. Government employee 3. Market trade vender 4. Daily laborer 5. Other (specify) _____________ |  |
| 106 | What is your husband’s current occupation? | 1. Farmer 2. Government employee 3. Daily laborer 4. Market trade vender 5. Other(specify) |  |
| 107 | Distance from health institution | 1. ----km |  |

| 1. **Information on obstetrics history** | | | |
| --- | --- | --- | --- |
| 201 | For how many times have you been pregnant (gravidity)? | ________ (enter number) | *Including current pregnancy ,abortion and stillbirths* |
| 202 | Are you pregnant now? | 1. Yes 2. No |  |
| 203 | Have you ever visited ANC follow up? | 1. Yes 2. no | (skip this question if she has no history of pregnancy ) |
| 204 | \| Did you give birth at health institution? \|  \| \| --- \| --- \| | 1. Yes 2. no |  |
| 205 | \| Have you ever used family planning service? \| \| --- \| | \| 1. yes 2.no \| \| --- \| |  |
| 03.Comprehensive knowledge of married women on HIV/AIDS and MTCT of HIV | | | |
| 301 | Have you ever heard about HIV/AIDS? | 1.yes 2.no |  |
| 302 | Ever tested for HIV? | 1.yes 2.no |  |
| 303 | What are the modes of HIV transmission? | 1. By unprotected sex with un infected person 2. By blood transfusion with infected blood 3. By sharing sharp instruments 4. By mosquito bite 5. Through MTCT | Encircle all the possible answer |
| 304 | Does HIV transmit from mother to child? | 1. 1.yes 2.no |  |
| 305 | If Yes for question number 3 When does HIV infected woman transmit the virus to her child? | 1. during pregnancy 2. during delivery 3. through breastfeeding | Encircle all the possible answer |
| 306 | Had discussion with your husband about HIV/AIDS? | 1.yes 2.no |  |
| 307 | Where have you heard this information above from? | 1.Health professionals  2. Friends/relatives  3. School  4. Mass media  *5. Others (specify)____* | Encircle all the possible answer |
