# Supplementary material for: Determinant factor of married women’s knowledge on vertical transmission of HIV in Mecha district, Ethiopia; a community-based study
Source: PLoS One. 2020 Dec 2;15(12):e0242659. doi: 10.1371/journal.pone.0242659 (PMC7710110; doi:10.1371/journal.pone.0242659)
Supplement: S2 File — (DOCX) [file pone.0242659.s002.docx]

አማርኛ መጠይቅ የመረጃና የስምምነት ውል ቅጽ

ሀ መግቢያ

ጤና ይሰጥልኝ ------------------------እባላለሁ፡፡በአሁን ሰዓት የጥናታዊ ጽሑፍ መረጃ በመሰብሰብ ላይ እገኛልሁ፡፡ እርስዎም የጥናቱ ተሳታፊ ይሆኑ ዘንድ ተመርጠዋል፡፡

**ዓላማ** ፡-በሜጫ ወረዳ ውስጥ የሚገኙ ባለትዳር ሴቶችን የኤችአይ ቪ ኤድስ በሽታ ክእናት ወደ ልጅ ስለመተላለፉ ያላቸውን እውቀት እና ተያያ ዥጉዳዮችን ለማወቅ የሚካሄድ ጥናትነው፡፡

**አተገባበር**፡ ጥናቱን ለማካሄድ ጥያቄዎች ይኖሩናል፡፡ ይህ ጥናት ተገባራዊ ሊሆን የሚችለው እርስዎ በሚሰጡት ትክክለኛ መልስ በመሆኑ ጥያቄዎቹን በጥንቃቄ እንዲመልሱልን በትህትና እንጠይቃለን፡፡እንዲብራራልወት የሚፈልጉት ጉዳይ ካለ መጠየቅ ይችላሉ፡፡ መጠይቁ 20 ደቂቃ አካባቢ የሚጨርስ መሆኑን ለመግለጽ እንወዳለን፡፡

**የጥናቱ ጠቀሜታ:-**

በዚህ ጥናት በመሳተፍዎ እርስዎ በቀጥታም ሆነ በተዘዋዋሪ ተጠቃሚ ሊሆኑ ይችላሉ፡፡ ስለዚህ በዚህ ጥናት ቢሳተፉ ባለትዳር ሴቶችን የኤችአይ ቪ ኤድስ በሽታ ክእናት ወደ ልጅ ስለመተላለፉ ያላቸውን እውቀት እና ተያያዥ ጉዳዮችን ለማወቅና ያሉትን የእውቀት ክፍተቶች ላማስተካከል በግብዓትነት ያገለግላል ብለን እናምናለን፡፡

**የመረጃ ምስጢራዊነት:-** ለዚህ ጥናት ስኬታማነት የሚሰጡት ማንኛውም መረጃ ምስጢራዊነቱ የተጠበቀና ስምዎትም የማይፃፍ ከመሆኑም ባሻገር መልስ የሰጡበትም ወረቀት የራሱ ኮድ ተሰጥቶትና ተቆልፎ የሚቀመጥ ነው::ጥናቱ ከተጠናቀቀ በኋላ ሁሉም የመጠየቂያ ወረቀቶች በእሳት ይቃጠላሉ፡፡

በዚህ ጥናት አለመሳተፍ ወይንም በማንኛውም ጊዜና ሁኔታ ማቋረጥም ሆነ ለአንዳንድ ጥያቄዎች መልስ አለመስጠት ይቻላሉ:: ለመሳተፍ ፋቃደኛ ባለመሆንዎም ምንም አይነት ተጽዕኖ እንደማይደርስብዎ ልናረጋግጥልዎ እንወዳለን::

በጥናቱ ለመሳተፍ ፈቃደኛ ነዎት? 1. አዎን ፊርማ--------------- 2. አይደለሁም

ለመሳተፍ ፍቃደኛ ከሆኑ አመስግነው ቃለ መጠይቁን ያካሂዱ፡፡ ፈቃደኛም ካልሆኑ አመስግነው ወደ ሌላ ተሳታፊ ይለፉ፡፡

ለበለጠ መረጃ ጥናት አጥኚዉን በሚከተለዉ አድራሻ ማግኘት ይችላሉ፡፡

ስም፡ተዋቸውሙጨ

ቃለ ምልልሱን ያካሄደው ስም…………………… ፊርማ------------------------ ቀን--------------

የተመራማሪው ስም፡ ጠዋቸው ሙጨ ፊርማ------------------------------------ቀን---------ስልክቁጥር፡0918475767

-ኢሜይል፡tewye2006@gmail.com

አማርኛ መጠይቅ

| O1. ማህበራዊ አና ስነ ህዝብ .መረጃዎች | | | |
| --- | --- | --- | --- |
| **ኮድ** | **ጥያቄዎች** | **መልስ** | **ምርመራ** |
| 101 | እድሜዎት ስንት ነው? | _________ | ሙሉ አመት |
| 102 | መኖሪያ ቦታ? | 1. ከተማ 2. ገጠር |  |
| 103 | የእርስዎ የትምህርት ደረጃ? | 1. ያልተማሩ 2. አንደኛ ደረጃ (ከ 1-8ኛ) 3. 2ኛ ደረጃ እና በላይ |  |
| 104 | የባለቤትዎ የትምህርተ ደረጃ? | 1. ያልተማሩ 2. አንደኛ ደረጃ (ከ 1-8ኛ) 3. 2ኛ ደረጃ እና በላይ |  |
| 105 | ስራዎት ምንድን ነው? | 1. የቤት አመቤት 2. የመንግስት ሰራተኛ 3. ነጋዴ 4. የቀን ሰራተኛ 5. ሌላ (የግለጹ) _____________ |  |
| 106 | የባለቤትዎ ስራ? | 1. ገበሬ 2. የመንግስት ሰራተኛ 3. የቀን ሰራተኛ 4. ነጋዴ 5. ሌላ (የግለጹ) _____________ |  |
| 107 | ከቤትዎ እስክ ጤና ተቋም ምን ያህል ይርቃል ? | --------ኪሜ |  |

| 1. ስነ-ተዋልዶ ጤና መረጃ | | | |
| --- | --- | --- | --- |
| 201 | ለስንት ጊዜ አርግዘው ያዉቃሉ | ________ | *ዉርጃን ጨምሮ* |
| 202 | አሁን ነፍሰጠር ነዎት | 1. አዎ 2. አይደለሁም |  |
| 203 | የነፍሰጡር ክትትል አድርገው ያውቃሉ | 1. አዎ 2. አላደረግሁም | አርግዘው ካላወቁ ይህ ጥያቄ ይዘለል |
| 204 | ጤና ተቋም ወልደዉ ያዉቃሉ | 1. አዎ 2. የለም |  |
| 205 | የቤተሰብ ምጣኔ ተቃሚ ሆነው ያውቃሉ? | 1. አዎ 2. የለም |  |
| 03 አጠቃላይ ስለ ኤች አይ ቪ ኤድስ በሽታ እውቅና ስለመኖር | | | |
| 301 | ስለ ኤች አይ ቪ ኤድስ በሽታ ሰምተው ያውቃሉ? | 1.አውቃለሁ 2.አላዉቅም |  |
| 302 | የኤች አይ ቪ ኤድስ ምርመራ አድርገው ያደርጋሉ? | 1.አውቃለሁ 2.አላዉቅም |  |
| 303 | አንድ ሰው እንዴት በኤች አይ ቪ ኤድስ በሽታ ሊያዝ ይችላል? | 1 በበሽታው ከተያዘ ሰው ጋር ጥንቃቄ የጎደለው የግብረ ስጋ ግንኙነት  2.በበሽታው ከተያዘ ሰው ደም በመቀበል  3.ከእናት ወደ ልጅ  4.ስለታማ ነገሮችን በጋራ በመተቀም  5. ሌላ (ይገለጽ)….. | በተሳታፊዎች የተጠቀሱት በሙሉ ይከበቡ |
| 304 | የኤች አይ ቪ ኤድስ በሽታ ክእናት ወደ ልጅ ይተላለፋል | 1.አዎ 2.አይደለም  3.አላዉቅም |  |
| 305 | የጥያቄ ቁጥር 402 መልስ አዎ ከሆነ እንዴት ሊተላለፍ ይችላል ይላሉ? | 1.በእርግዝና ጊዜ(ማህጸን ውስጥ እንዳለ)  2. በወሊድ ጊዜ  3.ጡት በሚጠባበት ጊዜ  4.አላዉቅም | በተሳታፊዎች የተጠቀሱት በሙሉ ይከበቡ |
| 306 | ከባለቤትዎ ጋር ስለኤችአይ ቪ ኤድስ ተወያይተው ያውቃሉ ? | 1.አዎ 2. አላውቅም |  |
| 307 | በአጠቃላይ መረጃዉን የት አገኙት(ስለ ኤች አይ ቪ ኤድስ በሽታ፣ መተላለፊ እናመከላከያ) | 1.ከጤና ባለሙያ  2. ከብዙሃን መገናጋ  3. ከጓደኛ/ዘመድ  4. ከትምህርት ቤት  *5.* ሌላ (ይገለጽ)….. | በተሳታፊዎች የተጠቀሱት በሙሉ ይከበቡ |
